# Treatment with radiosynoviorthesis in hemophilic patients with and without inhibitor

**DOI:** 10.1186/s12887-020-02071-3

**Published:** 2020-04-20

**Authors:** Mario Edgar Tena-Sanabria, Yoriko Fabiola Rojas-Sato, José Carlos Castañeda-Resendiz, Gabriela Fuentes-Herrera, Félix Alejandro Álvarez-Martínez, Yureni Iraí Tena-Gonzalez, Juan Carlos Núñez-Enríquez

**Affiliations:** 1 Orthopedics Department, UMAE Hospital de Pediatría “Dr. Silvestre Frenk Freund”, Centro Médico Nacional Siglo XXI, Instituto Mexicano del Seguro Social, Mexico City, Mexico; 2 Medical Research Unit in Clinical Epidemiology, UMAE Hospital de Pediatría “Dr. Silvestre Frenk Freund”, Centro Médico Nacional Siglo XXI, Instituto Mexicano del Seguro Social, Mexico City, Mexico

**Keywords:** Hemophilia. Hemarthrosis. Hemophilic arthropaty. Radiosynoviorthesis

## Abstract

**Background:**

Spontaneous bleedings occurring into joints (hemarthrosis) are the most common manifestations of hemophilia and causes severe joint damage ultimately resulting in joint disfunction known as hemophilic arthropathy. Among available therapeutic options for reducing recurrent hemarthrosis-associated damage, radiosynoviorthesis (RS) has proven effective in improving joint function.

**Aim:**

To assess the impact of RS with Yttrium(90) citrate (C-Y(90)) on frequency of hemarthroses and joint function in a group of pediatric patients.

**Methods:**

Between November 1998 and February 2017, we evaluated 27 pediatric patients with mild, moderate or severe hemophilia with haemophilic arthropathy. Overall, RS was applied in 60 joints. Some patients received more than one single intra-articular injection with C-Y(90).

**Results:**

During the follow-up, one patient showed joint bleeding 15 months after RS, one patient after 12 months and one patient after 45 days. The episodes of hemarthrosis were reduced and joint function significantly improved in all patients.

**Conclusion:**

RS with C-Y(90) is a simple and safe treatment for reducing the frequency of hemarthroses in patients with hemophilia. It decreases the use of factor VIII / IX and improves joint function.

## Background

Haemophilia A and B are X-linked coagulation disorders caused by the deficiency of factor VIII (FVIII) and factor IX (FIX), respectively. Almost one-third of hemophilic patients will develop inhibitors against FVIII / FIX during replacement therapy (more common in hemophilia A than in B), which increases the difficulty in treatment [1].

Haemophilia A affects 1 in 10,000 male live births, while hemophilia B affects 1 in 30,000 or more [1, 2]. In Mexico, the exact figure is unknown because there is still not a reliable national registry that allows us an accurate epidemiological diagnosis.

Spontaneous bleedings occurring into joints (hemarthrosis) are the most common manifestations of hemophilia, accounting for 65-80% of all bleedings in patients with severe hemophilia [3, 4]. Although any joint may be affected, the most frequent are knees, ankles, elbows, hips and shoulders, however, the frequency varies in different scenarios. For instance, knees are most affected in on demand patients, while ankles are the main sites involved in patients on prophylaxis. Recurrent bleeding from the synovial vessels into the intra-articular space facilitates iron deposits, which trigger a citokyne-mediated inflammatory and oxidative reaction, resulting in vascular proliferation. This vicious circle promotes synovial hypertrophy and predisposes to repetitive hemarthrosis in the same joint (target joint), which eventually lead to the functional disability known as hemophilic arthropathy [4–8].

In addition to its impact on the patient’s functional capacity, hemophilic arthropathy increases costs of hospitalization due to the time that patients must remain hospitalized, both during acute exacerbations and subsequent rehabilitation necessary to return to their normal daily activities.

Hematologic prophylaxis is recommended as mechanism to prevent bleedings and preserve musculoskeletal function. Haemophilic arthropathy can be prevented by giving regular prophylaxis and implementing physiotherapy programme, [4] but if chronic synovitis is already evident, despite the use of prophylaxis, it should be treated with chemical or radioisotope synoviorthesis [4, 9], with surgical procedures being restricted to non-responding patients to previous strategies.

The term synoviorthesis (medical synovectomy) is commonly used to describe the intra-articular procedures aimed to produce destruction/ fibrosis of the synovial membraneand subsynovial plexus.

Radiosynoviorthesis (RS) is a therapeutic modality based on the intra-articular injection of a colloidal suspension of radioisotope-labeled particles aimed to the restoration (orthesis) of the synovium.

Historically, this procedure has been performed with Yttrium(90) citrate (C-Y(90)), phosphor 32 (32P), gold 138 (138Au), renium 138 (138 Re) or erbium 169 (169 Er). New radioisotopes have now been developed, such as samarium 153 (153 Sm) and holmium 166 (166 Ho), which have shown similarly effective results for patients with chronic synovitis [4, 9].

The aim of our study was evaluating the efficacy of RS with C-Y(90) on joints with recurrent hemarthroses in pediatric patients with mild, moderate or severe hemophilia with and without inhibitor.

In particular, we tried to quantify the hemarthroses-free time and joint function improvement after the use of RS with C-Y(90) and to determine whether the use of factor VII, VIII and IX decreased in the emergency department.

## Methods

Patients with severe hemophilia treated with RS using C-Y(90) during November 1998 to February 2017 at the Department of Orthopedics of the Pediatric Hospital, National Medical Center Siglo XXI, IMSS were enrolled in the present study.

The variables collected included place of birth; age at the time of the first C-Y(90) application, weight and height; degree and stage of haemophilic arthropathy. The response of the joint after application of C-Y(90), the number of hemarthrosesper month, the number of doses of factor VIII, IX, and factor VII for patients with inhibitor, number of joints treated, number of patients with inhibitor, joint bleeding-free time and function were also recorded.

To classify the hemophilic arthropathyat each time of C-Y(90) application we used Pettersson score. This is based on typical findings of hemophilic arthropathy on posterior-anterior and lateral X-rays, including osteoporosis, enlargement of epiphysis, irregularity of subchondral surface, narrowing of joint space, subchondral cysts, erosions at joint margins, incongruence between joint surfaces, and the angulation and/or displacement of articulating bone ends. The maximum Pettersson score joint is 13 points indicating more joint damage [10].

The decision to use RS was based on the clinical status of patients at the moment of admission to our department which had been referred from second-level hospitals. None of the patients had received prophylactic treatment and presented recurrent joint bleedings (two or more joint bleeds in one month). They presented signs of progressive arthropathy (being no greater than grade III, according to the Arnold and Hilgartner classification), limited mobility arches and/or an increased joint size or pain.

For the RS, different radioisotopes can be used. The selection of the radioisotope should take into account two parameters [4, 11]: 1) physical properties: half-life, soft tissue penetration, size of the radiocolloid, 2) and clinical features: joint size, amount of joint fluid, synovial thickness. The material should also be a pure beta emitter, thereby minimizing the whole body exposure to gamma radiation [4, 8, 12, 13].

Among the available radioisotopes, we chose C-Y(90) taking into account their physical properties and clinical characteristics, as well as the good results obtained with RS with this medication published in the literature in pediatric patients.

All patients received replacement therapy to cover the procedure with FVIII and FIX except those with inhibitor. Of the group with inhibitor (n=6): one patient received activated recombinant FVII, while the others received FEIBA (mainly non-activated factors II, IX and X, as well as activated factor VII)".

After the application of C-Y(90), a cotton bandage (called Jones' bandage) was placed for three days, and rest with limb elevation at home was indicated; ambulation was allowed in those who presented hemophilia type A and B without inhibitor. The response to the procedure was evaluated by the presence / absence of bleeding of the treated joint, with monthly assessment, and then every three and six months during the follow-up time before being discharged from the Pediatric Unit.

Patients with inhibitor were admitted to the hospital for two or three days to administer the necessary medication and to control bleeding. Depending on the affected joint, the procedure, type of hemophilia, and its severity, substitution therapy was maintained.

## Results

During the study period, RS with C-Y(90) was applied in 60 joints of 27 patients: 29 knees, 21 ankles, and 10 elbows. The patients were between 3 and 16 years old at the time of the first RS procedure. They suffered between two and five episodes of hemarthrosis per month (12 per year in average) beforeC-Y(90)application. 22 had hemophilia A and 5 had hemophilia B. In the group with severe hemophilia A, five were patients with inhibitor (Table 1). Before RS treatment initiation and follow-up period none of the patients received prophylaxis.

**Table 1:** Clinical characteristics of Mexican children with hemophilia treated with Radiosynoviorthesis with Yttrium(90) citrate

Patient ID	Type of hemophilia	Grade	Inhibitor	Age (in years) at the time of the first RS with C-Y(90)	Average of bleeding events per year before treatment with C-Y(90)	Average of bleeding events per year after treatment with C-Y(90)	Joint treated with RS (1st application)	Pettersson score	Joint treated with RS (2nd application)	Pettersson score	Joint treated with RS (3rd application)	Pettersson score	Joint treated with RS (4th application)	Pettersson score
1	A	Moderate	No	10	8	0–1	Ankle	8	Elbow	8				
2	A	Severe	No	15	11	0–1	Knee/Ankle	8/3	Knee	8				
3	A	Mild	No	10	3	0–1	Knee	3						
4	A	Severe	No	14	10	0–1	Knee	8						
5	A	Severe	No	10	12	0–1	Knee	3	Knee/Ankle	8/8				
6	B	Severe	No	14	20	0–1	Knee	3	Knee	3				
7	A	Moderate	No	14	7	0–1	Ankle/Elbow	3/8	Knee	8	Knee	3	Knee	3
8	A	Severe	**Yes**	3	36	10	Knee	1	Ankle	3	Knee	8	Knee	8
9	A	Severe	**Yes**	11	18	0–1	Knee	3						
10	A	Severe	**Yes**	8	16	0–1	Ankle	8						
11	A	Severe	No	15	11	3	Knee	3	Knee	8	Knee	3		
12	A	Severe	**Yes**	11	30	0–1	Ankle	8	Ankle	8				
13	A	Severe	**Yes**	14	18	0–1	Ankle	8	Ankle	3	Knee	8		
14	A	Severe	No	11	10	0–1	Ankle	8	Ankle	8				
15	B	Moderate	No	9	11	0–1	Ankle/Elbow	3/1	Ankle	3				
16	A	Severe	No	14	11	0–1	Ankle	3	Ankle	3				
17	A	Moderate	No	9	6	0–1	Knee/Ankle	3/3						
18	A	Moderate	No	13	6	0–1	Knee	3						
19	A	Severe	No	15	12	0–1	Elbow	8						
20	A	Severe	No	13	12	0–1	Ankle/Elbow	3/8	Elbow	3				
21	A	Moderate	No	16	7	0–1	Knee	8						
22	A	Moderate	No	14	8	0–1	Knee	8						
23	A	Severe	**Yes**	13	15	0–1	Knee	8	Knee	3				
24	B	Severe	No	9	18	0–1	Knee/Elbow	8/8						
25	A	Severe	No	13	12	0–1	Elbow/Knee/Ankle	3/3/3	Ankle	3				
26	A	Severe	No	9	11	0–1	Ankle/Elbow	3/8						
27	A	Severe	No	15	10	3	Elbow	3	Knee	8	Knee	8		
*RS C-Y(90)* Radiosynoviorthesis with Yttrium(90) citrate														

The patients were followed in our center while they were pediatric (median 9.5 months). When they reached the age of 16 years and 11 months they were referred to be treated in adult units. The younger patients included in our study are currently under surveillance.

During the follow-up period, three patients presented bleeding after RS. The first patient, 15 years old, with severe A hemophilia and an average of 11 bleeding events per year before RS, suffered a first joint bleeding 15 months after RS, afterwards, had an average of three bleeding events per year (Table 1); the second patient, a 15 years old adolescent with severe A hemophilia without inhibitor, had his first bleeding at 12 months after RS, then, he had an annual average of three bleedings; the third patient was a three years old child diagnosed with severe A hemophilia and with inhibitor, he suffered the first bleeding after 45 days of RS treatment, and had an average of 10 bleeding events per year. This patient progressed to end-stage arthropathy. At the moment of the first event of bleeding in these patients, a new intra-articular injection with C-Y(90) was performed.

**Fig. 1:**
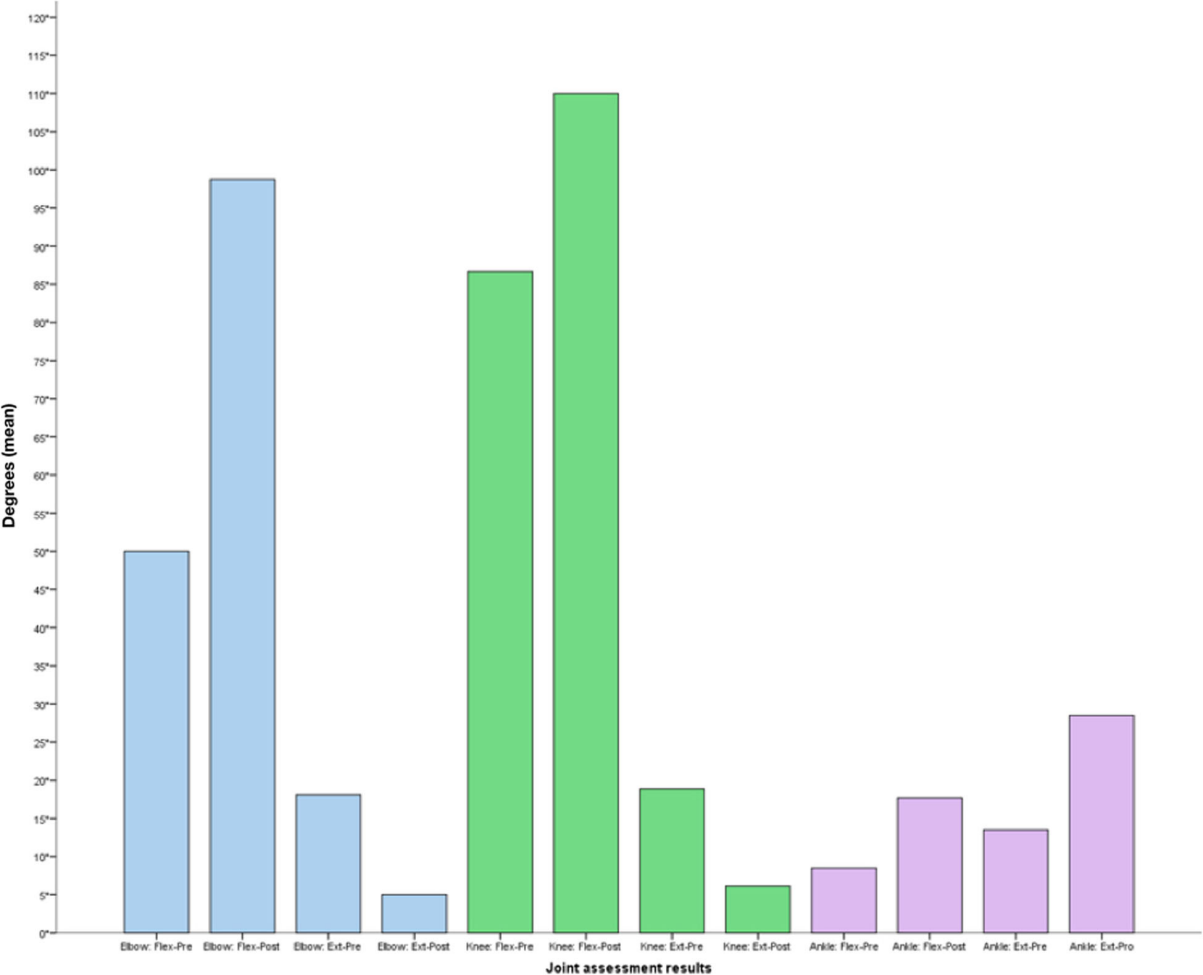
Results of the joint function assessment pre- and post-the last application of Yttrium(90) citrate

The remaining patients had no new hemarthrosis during the follow-up period. As patients did not present hemarthrosis, they recovered their mobility arches that were previously limited by joint bleedings (Fig. 1).

Four patients with inhibitor showed high response with previous administration of FVIIrA, suggesting that the presence of inhibitor does not prevent RS from being effective.

## Discussion

In the present study, the episodes of hemarthrosis were reduced and joint function significantly improved in all patients after RS.

In other investigations, it has been reported a reduction of inflammation in affected joints after the use of RS. Later, at two or three months, sclerosis and fibrosis of the synovial membrane have been observed [14].

RS in children is a controversial therapeutic procedure due to the use of radioactive isotopes. However, in line with our results, several studies have shown an excellent response to RS in children with hemophilia, and a good safety profile. Nonetheless, the number of children evaluated in the present research was limited, however, all those patients who met the selection criteria during study period were treated on demand with RS.

Research by Rodríguez-Merchán et al. in the hemophilia center of the Hospital de la Paz in Madrid, Spain, showed an improvement in joint function in young patients treated with RS in whom synovial membranes were not yet severely injured [15]. Heim et al. reported that RS in hemophiliac patients with chronic synovitis provides an 80% decrease in hemarthrosis and that 15% of cases did not present new episodes [16]. Manco-Johnson et al. in a study with 91 joints of 59 children with hemophilia with and without inhibitors of coagulation factors VIII and IX, found that RS limited the frequency of hemarthrosis, decreased pain, and improved joint function [17]. Kavakli et al in a series of 221 RS in 150 children and young adults reported a reduction of hemarthrosis in all patients, especially those with grade II synovitis and with early experience with 90Y [18]. Alioglu et al evidenced that RS was the best therapeutic procedure to halt the evolution of chronic hemophilic synovitis into hemophilic arthropathy in 18 patients with a mean age of 12 years and severe hemophilia with grade I or II synovitis [19]. Also Turkmen et al. in their study on the use of RS withY-90 on 82 knees of 67 pediatric patients and young adults with synovitis secondary to hemophilia showed that it was possible to delay the progression of arthropathy and increase the time to re-bleeding [20]. Martínez-Esteve et al. in their series of 20 children and adolescents with hemophilia (mean age 13 years) reported that in the 70.3% of the joints the RS had a good or excellent response and in 29.7% a partial response [21].

The efficacy of RS ranges between 76% and 80%, and the percentage of failure of the procedure in the control of hemarthrosis is considered to be 20%. When compared with chemical synoviorthesis, authors such as Bessant et al. report a variable and changing response throughout the months of evolution, with efficacy being 67% at six months and 50% at twelve months [22].

Complications are rare if the technique is performed properly. General complications include fever or allergy to the radiotracer (very rare), and local complications, related to joint puncture, include local pain, septic arthritis or lymphedema. Cutaneous necrosis with fistulation through the needle path is very rare. There is a risk of acute synovitis after treatment, although this possibility decreases when corticosteroids are associated. Premalignant lesions or changes in the structure of chromosomes have not been demonstrated in patients who have been given 90Y and 186 Re [23]. In the present study, neither adverse effects or complications associated with RS usingC-Y(90) were observed.

The effectiveness of RS, the decreased number of procedures required for controlling hemarthrosis, the improvement in joint mobility and the lower amount of prophylaxis required are parameters that all together have led to recommend RS as the procedure of choice in patients with haemophilic arthropathy. It has been mentioned that RS presents a number of advantages such as: simplicity, safety, shorter or unnecessary hospitalization, limited cost, as well as the possibility of repeating the technique without loss of efficacy and performing posterior surgical synovectomy, if necessary [16–21, 24]. In the present research, emergency service use was reduced. Presumably, but not evaluated in the present investigation, need for prophylaxis, costs of hospitalization were reduced in all patients both during acute joint bleedings and subsequent rehabilitation therapy necessary to return to their normal daily activities, including school attendance.

## Conclusion

Radiosynoviorthesis with C-Y(90) may be the ideal treatment for recurrent hemarthrosis in patients with hemophilia, considering that it reduces inflammation and, later on (two to three months), causes sclerosis and fibrosis of the synovial membrane, thus avoiding new episodes of hemarthrosis.

In hemophilic arthropathy, the effectiveness of RS is reflected in the decrease in the number of procedures required to control hemarthrosis. It is used in a single application, the procedure is simple, safe, of limited cost, and can be repeated if necessary. It does not require hospital admission, it has an improvement in joint mobility and a decrease in the use of factor VIII / IX, it does not cause adverse reactions such as sarcomas or leukemias and represents a significant saving for the institution. In our opinion, these advantages lead to the recommendation of radiosynoviorthesis as the procedure of choice in patients with hemophilic arthropathy.

## Data Availability

Not applicable.

## Abbreviations

RS: Radiosynoviorthesis
C-Y(90): Yttrium(90) citrate
32P: Phosphor 32
138Au: Gold 138
138 Re: Renium 138
169 Er: Erbium 169
153 Sm: Samarium 153
166 Ho: Holmium 166

## References

[1] Mannucci PM, Tuddenham EG (2001). The hemophilias - from royal genes to gene therapy. N Engl J Med.

[2] Report on the World Federation of Hemophilia Annual Global Survey. 2018. https://www.wfh.org/en/our-work-research-data/annual-global-survey. Accessed 10 Nov 2019.

[3] Coppola A, Franchini M (2013). Target of prophylaxis in severe haemophilia: more than factor levels. Blood Transfus.

[4] Pasta G, Mancuso ME, Perfetto OS, Solimero LP (2009). Radiosynoviorthesis in children with haemophilia. Hämostaseologie.

[5] Aronstam A, Rainsford SG, Painter MJ (1979). Patterns of bleeding in adolescents with severe haemophilia a. BMJ.

[6] Fernandez-Palazzi F (1998). Treatment of acute and chronic synovitis by non-surgical means. Haemophilia.

[7] Chrapko B, Zwolak R, Nocuń A, Gołębiewska R, Majdan M (2007). Radiation synovectomy with 90Y colloid in the therapy of recurrent knee joint effusions in patient with inflammatory joint diseases. RheumatolInt.

[8] Rodriguez-Merchan EC, Goddard NJ (2001). The technique of synoviorthesis. Haemophilia.

[9] Heim M, Horoszowski H, Lieberman L, Varon D, Martino-witz U, Gilbert MS, Greene WD (1990). Methods and results of radionucleotide synovectomies. Musculoskeletal Problems in Hemophilia.

[10] Pettersson H, Ahlberg A, Nilsson IM (1980). A radiologic classification of hemophilic arthropathy. Clin OrthopRelat Res.

[11] Mödder G (1995). Die Radiosynoviorthese.

[12] LLJ H-D, HCW DV, van der Linden S (2000). Yttrium radiosynoviorthesisen the treatment of knee arthritis in rheumatoid, arthritis:a systematic review. Ann Rheum Dis.

[13] RodriguezMerchan EC, Quintana M (2007). De la Corte Rodriguez. Radioactive synoviorthesis for the treatment of haemophilicsinovitis. Haemophilia.

[14] Özcan Z (2014). Radiosynovectomy in hemophilic Synovitis. Mol Imaging Radionuclide Therapy.

[15] Rodriguez-Merchan EC, Caviglia HA, Magallon M, Perez-Blanco R (1997). Chemical synovectomy vs. radioactive synovectomy for the treatment of chronic haemophilic synovitis: a prospective short-term study. Haemophilia.

[16] Heim M, Tiktinsky R, Amit Y, Martinowitz U (2004). Yttrium synoviorthesis of the elbow joints in persons with haemophilia. Haemophilia.

[17] Manco-Johnson MJ, Nuss R, Lear J (2002). 32P Radiosynoviorthesis in children with hemophilia. J PediatrHematol Oncol.

[18] Kavakli K, Aydogdu S, Taner M (2008). Radioisotope synovectomy with rhenium186 in haemophilic synovitis for elbows, ankles and shoulders. Haemophilia.

[19] Alioglu B, Ozsoy H, Koca G, Sakaogullari A, Selver B, Ozdemir M (2010). The effectiveness of radioisotope synovectomy for chronic synovitis in Turkish paediatrichaemophiliacs: Ankara experience. Haemophilia.

[20] Turkmen C, Kilicoglu O, Dikici F, Bezgal F, Kuyumcu S, Gorgum O (2014). Survival analysis of Y-90 radiosynovectomy in the treatment of haemophilic synovitis of the knee: a 10-year retrospective review. Haemophilia.

[21] Martínez-Esteve A, Álvarez-Pérez RM, Núñez-Vázquez R (2016). Sinoviortesisradioisotópica en pacientes en edad pediátrica y adolescentes con hemofilia. Rev Esp Med Nucl Imagen Mol.

[22] Bessant R, Steuer A, Rigby S, Gumpel M (2003). Osmic acid revisited: factors that predict a favourable response. Rheumatology (Oxford).

[23] Vuorela J, Sokka T, Pukkala E, Hannonen P (2003). Does yttrium radiosynovectomy increase the risk of cancer in patients with rheumatoid arthritis?. Ann Rheum Dis.

[24] Rodriguez-Merchan EC, De la Corte-Rodriguez H, Jimenez-Yuste V (2014). Radiosynovectomy in haemophilia: long-term results of 500 procedures performed in a 38-year period. Thromb Res.

